# Triethyl­ammonium hexa-μ_2_-acetato-κ^12^ *O*:*O*′-diacetato-κ^2^ *O*-aqua-μ_3_-oxido-triferrate(III) toluene monosolvate

**DOI:** 10.1107/S160053681102616X

**Published:** 2011-07-13

**Authors:** Andrew R. Burgoyne, Reinout Meijboom, Alfred Muller, Bernard O. Omondi

**Affiliations:** a Research Centre for Synthesis and Catalysis, Department of Chemistry, University of Johannesburg, PO Box 524 Auckland Park, Johannesburg, 2006, South Africa

## Abstract

The title compound, (C_6_H_16_N)[Fe_3_(CH_3_CO_2_)_8_O(H_2_O)]·C_7_H_8_, was serendipitously crystallized from a reaction of disilanol with iron(II) acetate. The trinuclear acetatoferrate(III) anion has a triethyl­ammonium cation as the counterion. The three Fe atoms lie on the vertices of a regular triangle and are octa­hedrally coordinated. The complete coordination of the anion includes shared ligands among the three metal ions: a central tribridging O atom and six bidentate bridging acetyl groups. The six-coordinations of two of the metal ions are completed by a monodentate acetate ligand, whereas that of the third metal ion is completed by a water mol­ecule. The uncoordinated triethyl­ammonium cation is involved in N—H⋯O hydrogen bonding to a singly coordinated acetyl group. The coordinated aqua mol­ecule is involved in bifurcated O—H⋯O hydrogen bonding. C—H⋯O inter­actions are also observed. The toluene solvent molecule is disordered over two sets of sites in a 0.609 (11):0.391 (11) ratio.

## Related literature

For exchange-coupled structural fragments or exchange clusters in coordination chemistry, see: Cannon & White (1988 ▶). For applications and biological activity of 3*d*-element carboxyl­ates, see: Cannon & White (1988 ▶); West (1989 ▶); Muettertis (1981 ▶). For poly-iron carboxyl­ates, see: Crichton (1991 ▶). For bidentate *syn*–*syn* bridges, see: Porai-Koshits (1981 ▶). For related tri­oxy-bridged iron compounds, see: Turte *et al.* (2002 ▶). For the synthesis and characterization of iron carboxylate complexes, see: Losada *et al.* (1997 ▶); Rardin *et al.* (1992 ▶). 
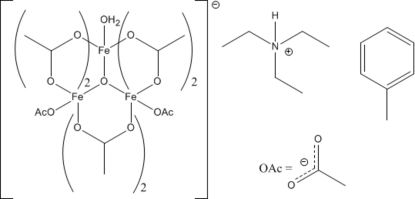


## Experimental

### 

#### Crystal data

(C_6_H_16_N)[Fe_3_(C_2_H_3_O_2_)_8_O(H_2_O)]·C_7_H_8_
*M* *_r_* = 868.25
Monoclinic, 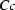

*a* = 22.4370 (11) Å
*b* = 11.1060 (5) Å
*c* = 16.5720 (9) Å
β = 112.904 (1)°
*V* = 3803.9 (3) Å^3^
*Z* = 4
Mo *K*α radiation
μ = 1.20 mm^−1^
*T* = 100 K
0.18 × 0.07 × 0.06 mm

#### Data collection

Bruker KappaCCD APEX DUO 4K diffractometer
Absorption correction: multi-scan (*SADABS*; Bruker, 2007 ▶) *T*_min_ = 0.813, *T*_max_ = 0.931
10304 measured reflections
7041 independent reflections
6362 reflections with *I* > 2σ(*I*)
*R*_int_ = 0.029

#### Refinement

*R*[*F* ^2^ > 2σ(*F* ^2^)] = 0.040
*wR*(*F* ^2^) = 0.099
*S* = 1.01
7041 reflections
543 parameters
159 restraints
H atoms treated by a mixture of independent and constrained refinement
Δρ_max_ = 0.89 e Å^−3^
Δρ_min_ = −0.40 e Å^−3^
Absolute structure: Flack (1983 ▶), 2220 Friedel pairs
Flack parameter: 0.341 (17)

### 

Data collection: *APEX2* (Bruker, 2007 ▶); cell refinement: *SAINT-Plus* (Bruker, 2007 ▶); data reduction: *SAINT-Plus* and *XPREP* (Bruker, 2007 ▶); program(s) used to solve structure: *SHELXS97* (Sheldrick, 2008 ▶); program(s) used to refine structure: *SHELXL97* (Sheldrick, 2008 ▶); molecular graphics: *DIAMOND* (Brandenburg & Putz, 2005 ▶); software used to prepare material for publication: *WinGX* (Farrugia, 1999 ▶).

## Supplementary Material

Crystal structure: contains datablock(s) I, global. DOI: 10.1107/S160053681102616X/kp2323sup1.cif

Structure factors: contains datablock(s) I. DOI: 10.1107/S160053681102616X/kp2323Isup2.hkl

Additional supplementary materials:  crystallographic information; 3D view; checkCIF report

## Figures and Tables

**Table 1:** Selected bond lengths (Å)

Fe1—O1	2.026 (3)
Fe3—O2	2.031 (3)
Fe1—O3	2.030 (3)
Fe3—O4	2.012 (3)
Fe3—O5	2.017 (3)
Fe2—O6	2.021 (3)
Fe2—O7	2.032 (3)
Fe3—O8	2.017 (3)
Fe2—O9	2.028 (3)
Fe1—O10	2.026 (3)
Fe2—O11	2.018 (4)
Fe1—O12	2.029 (3)
Fe2—O13	2.027 (3)
Fe3—O15	1.995 (3)
Fe1—O17	2.045 (3)
Fe1—O18	1.906 (3)
Fe2—O18	1.924 (3)
Fe3—O18	1.945 (3)

**Table 2:** Hydrogen-bond geometry (Å, °)

*D*—H⋯*A*	*D*—H	H⋯*A*	*D*⋯*A*	*D*—H⋯*A*
N1—H1⋯O14^i^	0.93	1.87	2.789 (6)	168
O17—H10⋯O16^ii^	0.83 (2)	1.81 (2)	2.635 (5)	176 (5)
O17—H11⋯O14^iii^	0.83 (2)	1.83 (2)	2.665 (4)	178 (5)
C12—H12*B*⋯O7^iv^	0.98	2.55	3.526 (6)	174
C14—H14*C*⋯O6	0.98	2.54	3.166 (7)	122
C15—H15*C*⋯O8	0.98	2.53	3.188 (6)	125
C19—H19*A*⋯O16^v^	0.99	2.37	3.221 (6)	144
C21—H21*B*⋯O15^v^	0.99	2.55	3.418 (5)	146
C22—H22*A*⋯O3^vi^	0.98	2.55	3.476 (6)	157

Symmetry codes: (i) 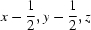; (ii) 
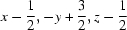
; (iii) 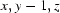; (iv) 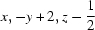; (v) 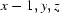; (vi) 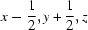.

**Fig. 1.**
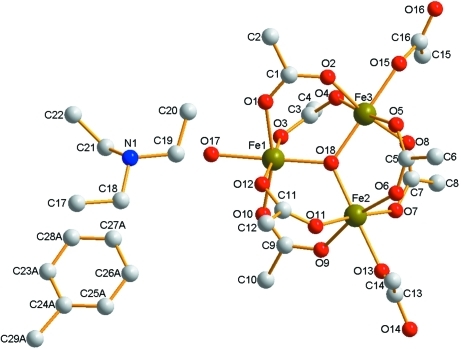
The asymmteric unit of (I) with structure of the 3-iron centre anion showing 30% probability displacement ellipsoids.

## supplementary crystallographic information

### Comment

Coordination chemistry has a large interest in exchange-coupled structural fragments or exchange clusters (Cannon and White, 1988). These compounds include complexes with carboxylic acids which are used as bridging anions, as shown in this title compound. Many 3*d*-element carboxylates have a variety of applications, including: catalysis, materials science and biological activity (Cannon and White, 1988; West, 1989; Muettertis, 1981). Poly-iron carboxylates as explained by Crichton (Crichton, 1991) (ferritin), have been shown to be useful in all these fields.

The title compound, Triethylammonium (µ^3^-oxo)-hexakis(µ^2^-acetato-O,O')-aqua-(diacetato-O) -tri-iron(iii) toluene solvate, serendipitously crystallized while trying to synthesize Fe-containing poly-oligomeric silasilsesquioxane material. The three Fe^3+^ are all inter-connected by bridging ligands, revealing distorted octahedral geometries (Table 1). The three Fe^3+^ are equally spaced around the central µ_3_-O_18_, with angles of: Fe_1_—O_18_—Fe_2_ 119.56 (17) °, Fe_1_—O_18_—Fe_3_ 119.80 (16) °, Fe_2_—O_18_—Fe_3_ 120.64 (17)°. The sum of the three angles around O_18_ is 360°. The three Fe^3+^  lie at the vertices of a virtual regular triangle. The Fe_1_—Fe_2_ distance is 3.305 Å, Fe_2_—Fe_3_ distance is 3.362 Å and Fe_1_—Fe_3_ distance is 3.334 Å. This is in accordance with related tri-oxy bridged iron compounds (Turte *et al.*, 2002). Six acetate ions act as bidentate *syn*-*syn*-bridges (Porai-Koshits, 1981) linking the iron atoms in pairs into the cluster. The Fe—O distances range from 2.012 (4) – 2.037 (4) Å. The uncoordinated triethylammonium  cation is involved in hydrogen bonding *via* N_1_—H_1_···O_14_ in which it is bonded to a singly coordinated acetyl group (symmetry code: *x* - 1/2, *y* - 1/2, *z*). The coordinated aqua molecule is involved in bifurcated hydrogen bonding, where O_17_—H_10_···O_16_ (symmetry code: *x* - 1/2, -*y* + 3/2, *z* - 1/2) and O_17_—H_11_···O_14_ (symmetry code: *x*, *y* - 1, *z*) are the 2 inter-molecular hydrogen bonds connecting the 2 H atoms from the coordinated aqua molecule to singly coordinated acetyl groups (Table 2).

### Experimental

The disilanol, 1,3,5,7,9-octaisobutyltetracyclooctasiloxane-*endo*-3,7-diol, (0.7 g, 0.785 mmol) was dissolved in toluene and reacted with triethyl amine for 20 minutes at room temperature under nitrogen. Then Fe(OAc)_2_ (0.273 g, 0.785 mmol) (OAc = acetate) was added to a stirring solution and left to react for 6 days. In a solution a red precipitate was formed which was filtered off and left to form crystals of the title compound in the toluene solvent.

### Refinement

All hydrogen atoms were positioned geometrically with C—H = 0.99 Å for methylene H atoms, 0.98 Å for methyl H atoms, 0.95 Å for aromatic H atoms, and 0.93 Å for N—H. All hydrogen atoms were allowed to ride on their parent atoms with *U*_iso_(H) = 1.2*U*_eq_. The toluene molecule showed large ellipsoids and thus was treated as a disordered species during the refinement. This resulted in population 0.61:0.39 over the two disordered positions. A slightly high residual electron density of 0.89 e.Å^-3^ located at 0.48 Å from H_28 A_. This is in the region of the toluene disorder and probably represents no physical meaning. Initial refinement cycles showed a non-zero Flack parameter. It was decided in subsequent refinement cycles to include racemic twinning of the compound. This refined to a 34.1 : 65.9 racemic twin. The two H atoms in the coordinated aqua molecule (O—H) were placed at constrained distances from a Fourier difference map.

### Crystal data

**Table:**

(C_6_H_16_N)[Fe_3_(C_2_H_3_O_2_)_8_O(H_2_O)]·C_7_H_8_	*F*(000) = 1812
*M**_r_* = 868.25	*D*_x_ = 1.516 Mg m^−^^3^
Monoclinic, *C**c*	Mo *K*α radiation, λ = 0.71073 Å
Hall symbol: C -2yc	Cell parameters from 10304 reflections
*a* = 22.4370 (11) Å	θ = 2.0–28.5°
*b* = 11.1060 (5) Å	µ = 1.20 mm^−^^1^
*c* = 16.5720 (9) Å	*T* = 100 K
β = 112.904 (1)°	Block, red
*V* = 3803.9 (3) Å^3^	0.18 × 0.07 × 0.06 mm
*Z* = 4	

### Data collection

**Table:**

Bruker KappaCCD APEX DUO 4K diffractometer	7041 independent reflections
Radiation source: fine-focus sealed tube	6362 reflections with *I* > 2σ(*I*)
graphite	*R*_int_ = 0.029
φ and ω scans	θ_max_ = 28.5°, θ_min_ = 2.0°
Absorption correction: multi-scan (*SADABS*; Bruker, 2007)	*h* = −30→29
*T*_min_ = 0.813, *T*_max_ = 0.931	*k* = −14→14
10304 measured reflections	*l* = −18→22

### Refinement

**Table:**

Refinement on *F*^2^	Secondary atom site location: difference Fourier map
Least-squares matrix: full	Hydrogen site location: inferred from neighbouring sites
*R*[*F*^2^ > 2σ(*F*^2^)] = 0.040	H atoms treated by a mixture of independent and constrained refinement
*wR*(*F*^2^) = 0.099	*w* = 1/[σ^2^(*F*_o_^2^) + (0.0492*P*)^2^ + 4.8845*P*] where *P* = (*F*_o_^2^ + 2*F*_c_^2^)/3
*S* = 1.01	(Δ/σ)_max_ = 0.001
7041 reflections	Δρ_max_ = 0.89 e Å^−^^3^
543 parameters	Δρ_min_ = −0.40 e Å^−^^3^
159 restraints	Absolute structure: Flack (1983), 2220 Friedel pairs
Primary atom site location: structure-invariant direct methods	Flack parameter: 0.341 (17)

### Special details

**Table:**

Experimental. The intensity data was collected on a Bruker *APEX* Duo 4 K KappaCCD diffractometer using an exposure time of 20 s/frame. A total of 589 frames were collected with a frame width of 0.5° covering up to θ = 28.5° with 99.3% completeness accomplished.
Geometry. All e.s.d.'s (except the e.s.d. in the dihedral angle between two l.s. planes) are estimated using the full covariance matrix. The cell e.s.d.'s are taken into account individually in the estimation of e.s.d.'s in distances, angles and torsion angles; correlations between e.s.d.'s in cell parameters are only used when they are defined by crystal symmetry. An approximate (isotropic) treatment of cell e.s.d.'s is used for estimating e.s.d.'s involving l.s. planes.
Refinement. Refinement of *F*^2^ against ALL reflections. The weighted *R*-factor *wR* and goodness of fit *S* are based on *F*^2^, conventional *R*-factors *R* are based on *F*, with *F* set to zero for negative *F*^2^. The threshold expression of *F*^2^ > σ(*F*^2^) is used only for calculating *R*-factors(gt) *etc*. and is not relevant to the choice of reflections for refinement. *R*-factors based on *F*^2^ are statistically about twice as large as those based on *F*, and *R*- factors based on ALL data will be even larger.

### Fractional atomic coordinates and isotropic or equivalent isotropic displacement parameters (Å^2^)

**Table:**

	*x*	*y*	*z*	*U*_iso_*/*U*_eq_	Occ. (<1)
C1	0.99287 (19)	0.7027 (4)	0.7988 (3)	0.0186 (8)	
C2	1.0241 (2)	0.6087 (4)	0.7635 (4)	0.0331 (12)	
H2A	1.0288	0.6395	0.7108	0.050*	
H2B	0.9971	0.5362	0.7486	0.050*	
H2C	1.0669	0.5890	0.8079	0.050*	
C3	0.9372 (2)	0.7163 (4)	0.9869 (3)	0.0231 (9)	
C4	0.9357 (3)	0.6701 (6)	1.0713 (4)	0.0465 (15)	
H4A	0.9678	0.6059	1.0945	0.070*	
H4B	0.8925	0.6382	1.0606	0.070*	
H4C	0.9457	0.7359	1.1139	0.070*	
C5	1.0197 (2)	1.1325 (4)	0.8475 (3)	0.0209 (8)	
C6	1.0602 (2)	1.2224 (4)	0.8224 (4)	0.0315 (11)	
H6A	1.0345	1.2560	0.7645	0.047*	
H6B	1.0984	1.1820	0.8206	0.047*	
H6C	1.0738	1.2874	0.8658	0.047*	
C7	0.96959 (19)	1.0670 (4)	1.0409 (3)	0.0211 (8)	
C8	0.9799 (3)	1.0896 (6)	1.1351 (4)	0.0477 (15)	
H8A	0.9407	1.1252	1.1377	0.072*	
H8B	1.0164	1.1449	1.1616	0.072*	
H8C	0.9894	1.0132	1.1672	0.072*	
C9	0.76697 (19)	0.9498 (4)	0.8365 (3)	0.0203 (8)	
C10	0.7001 (2)	0.9533 (4)	0.8387 (4)	0.0326 (11)	
H10A	0.6685	0.9780	0.7812	0.049*	
H10B	0.6998	1.0111	0.8832	0.049*	
H10C	0.6889	0.8732	0.8531	0.049*	
C11	0.8355 (2)	0.9855 (4)	0.6650 (3)	0.0234 (9)	
C12	0.8142 (3)	1.0167 (5)	0.5696 (4)	0.0397 (12)	
H12A	0.7719	0.9802	0.5366	0.060*	
H12B	0.8459	0.9859	0.5474	0.060*	
H12C	0.8110	1.1044	0.5625	0.060*	
C13	0.8422 (2)	1.3520 (4)	0.7996 (3)	0.0247 (9)	
C14	0.8671 (3)	1.3651 (5)	0.7282 (4)	0.0436 (15)	
H14A	0.8576	1.4463	0.7034	0.065*	
H14B	0.8460	1.3057	0.6822	0.065*	
H14C	0.9140	1.3519	0.7524	0.065*	
C15	1.1213 (2)	0.8291 (5)	1.1531 (3)	0.0304 (10)	
H15A	1.1578	0.8154	1.2089	0.046*	
H15B	1.0911	0.7613	1.1407	0.046*	
H15C	1.0989	0.9035	1.1567	0.046*	
C16	1.1456 (2)	0.8400 (4)	1.0815 (3)	0.0228 (9)	
O1	0.93335 (13)	0.6928 (2)	0.7810 (2)	0.0213 (6)	
O2	1.02872 (13)	0.7852 (3)	0.8441 (2)	0.0206 (6)	
O3	0.88558 (14)	0.7114 (3)	0.9197 (2)	0.0244 (7)	
O4	0.98994 (14)	0.7581 (3)	0.9902 (2)	0.0239 (7)	
O5	1.04519 (13)	1.0320 (3)	0.8760 (2)	0.0205 (6)	
O6	0.96459 (14)	1.1651 (3)	0.8409 (3)	0.0275 (8)	
O7	0.91988 (15)	1.1103 (3)	0.9815 (2)	0.0241 (7)	
O8	1.01182 (13)	1.0047 (3)	1.0281 (2)	0.0227 (6)	
O9	0.80089 (13)	1.0436 (2)	0.8579 (2)	0.0218 (6)	
O10	0.78355 (14)	0.8548 (3)	0.8106 (3)	0.0311 (8)	
O11	0.84602 (16)	1.0705 (3)	0.7181 (2)	0.0248 (7)	
O12	0.84056 (16)	0.8751 (3)	0.6845 (2)	0.0279 (7)	
O13	0.84926 (14)	1.2543 (3)	0.8403 (2)	0.0232 (7)	
O14	0.81293 (16)	1.4405 (3)	0.8161 (3)	0.0304 (8)	
O15	1.10608 (13)	0.8655 (3)	1.0038 (2)	0.0197 (6)	
O16	1.20332 (15)	0.8192 (4)	1.0977 (3)	0.0376 (9)	
O17	0.80861 (14)	0.6542 (3)	0.7410 (2)	0.0234 (7)	
Fe1	0.86677 (3)	0.79541 (5)	0.80355 (4)	0.01476 (12)	
Fe2	0.88626 (2)	1.08593 (5)	0.84973 (4)	0.01472 (12)	
Fe3	1.01309 (2)	0.89754 (5)	0.93051 (4)	0.01390 (12)	
H10	0.7746 (14)	0.663 (5)	0.698 (2)	0.021*	
H11	0.810 (2)	0.588 (3)	0.765 (3)	0.021*	
N1	0.2558 (2)	0.8833 (4)	0.9336 (3)	0.0353 (10)	
H1	0.2764	0.9131	0.8987	0.042*	
C17	0.1556 (3)	0.8770 (6)	0.8004 (5)	0.0519 (16)	
H17A	0.1322	0.9350	0.8217	0.078*	
H17B	0.1246	0.8234	0.7574	0.078*	
H17C	0.1808	0.9202	0.7728	0.078*	
C18	0.2000 (3)	0.8046 (5)	0.8752 (4)	0.0440 (14)	
H18A	0.2172	0.7357	0.8530	0.053*	
H18B	0.1761	0.7724	0.9096	0.053*	
C19	0.3046 (3)	0.8053 (5)	1.0036 (4)	0.0401 (13)	
H19A	0.2855	0.7777	1.0451	0.048*	
H19B	0.3143	0.7332	0.9757	0.048*	
C20	0.3647 (4)	0.8699 (6)	1.0519 (5)	0.0566 (17)	
H20A	0.3800	0.9090	1.0104	0.085*	
H20B	0.3976	0.8130	1.0882	0.085*	
H20C	0.3570	0.9310	1.0894	0.085*	
C21	0.2340 (2)	0.9891 (5)	0.9700 (4)	0.0398 (13)	
H21A	0.2587	0.9917	1.0341	0.048*	
H21B	0.1877	0.9787	0.9593	0.048*	
C22	0.2426 (3)	1.1085 (5)	0.9306 (4)	0.0407 (13)	
H22A	0.2883	1.1199	0.9412	0.061*	
H22B	0.2281	1.1745	0.9578	0.061*	
H22C	0.2168	1.1081	0.8674	0.061*	
C23A	0.0763 (5)	0.3095 (10)	0.0354 (9)	0.042 (3)	0.609 (11)
H23A	0.0461	0.2455	0.0146	0.050*	0.609 (11)
C24A	0.0575 (5)	0.4243 (10)	0.0090 (7)	0.045 (2)	0.609 (11)
C25A	0.0999 (6)	0.5198 (11)	0.0431 (10)	0.050 (3)	0.609 (11)
H25A	0.0866	0.5999	0.0247	0.060*	0.609 (11)
C26A	0.1613 (5)	0.4974 (9)	0.1039 (9)	0.046 (3)	0.609 (11)
H26A	0.1900	0.5624	0.1293	0.055*	0.609 (11)
C27A	0.1810 (4)	0.3809 (8)	0.1278 (7)	0.041 (2)	0.609 (11)
H27A	0.2237	0.3655	0.1688	0.049*	0.609 (11)
C28A	0.1387 (5)	0.2856 (9)	0.0922 (8)	0.044 (2)	0.609 (11)
H28A	0.1527	0.2049	0.1069	0.052*	0.609 (11)
C29A	−0.0089 (7)	0.4397 (18)	−0.0584 (12)	0.105 (6)	0.609 (11)
H29A	−0.0124	0.3984	−0.1123	0.157*	0.609 (11)
H29B	−0.0179	0.5257	−0.0705	0.157*	0.609 (11)
H29C	−0.0402	0.4053	−0.0369	0.157*	0.609 (11)
C23B	0.0310 (7)	0.3495 (12)	0.0090 (11)	0.049 (4)	0.391 (11)
H23B	−0.0092	0.3083	−0.0080	0.059*	0.391 (11)
C24B	0.0322 (8)	0.4684 (13)	−0.0103 (12)	0.049 (4)	0.391 (11)
H24B	−0.0068	0.5090	−0.0441	0.058*	0.391 (11)
C25B	0.0908 (8)	0.5305 (15)	0.0196 (16)	0.056 (6)	0.391 (11)
H25B	0.0926	0.6130	0.0055	0.067*	0.391 (11)
C26B	0.1459 (9)	0.4693 (13)	0.0702 (14)	0.049 (4)	0.391 (11)
H26B	0.1856	0.5123	0.0930	0.058*	0.391 (11)
C27B	0.1464 (8)	0.3473 (13)	0.0897 (12)	0.041 (3)	0.391 (11)
C28B	0.0881 (10)	0.2889 (15)	0.0531 (19)	0.055 (6)	0.391 (11)
H28B	0.0871	0.2038	0.0583	0.066*	0.391 (11)
C29B	0.2042 (9)	0.2809 (18)	0.1403 (12)	0.060 (5)	0.391 (11)
H29D	0.1933	0.1959	0.1428	0.091*	0.391 (11)
H29E	0.2231	0.3137	0.1998	0.091*	0.391 (11)
H29F	0.2355	0.2878	0.1126	0.091*	0.391 (11)
O18	0.92188 (12)	0.9263 (2)	0.86103 (19)	0.0146 (5)	

### Atomic displacement parameters (Å^2^)

**Table:**

	*U*^11^	*U*^22^	*U*^33^	*U*^12^	*U*^13^	*U*^23^
C1	0.0153 (17)	0.0218 (19)	0.016 (2)	0.0051 (15)	0.0031 (15)	0.0016 (16)
C2	0.022 (2)	0.030 (2)	0.049 (3)	0.0026 (19)	0.016 (2)	−0.013 (2)
C3	0.023 (2)	0.025 (2)	0.021 (2)	−0.0021 (17)	0.0072 (18)	0.0010 (18)
C4	0.037 (3)	0.072 (4)	0.024 (3)	−0.023 (3)	0.004 (2)	0.009 (3)
C5	0.0202 (19)	0.0209 (19)	0.024 (2)	−0.0015 (16)	0.0116 (18)	0.0011 (17)
C6	0.024 (2)	0.027 (2)	0.050 (3)	−0.0002 (18)	0.022 (2)	0.012 (2)
C7	0.0173 (18)	0.023 (2)	0.021 (2)	−0.0024 (16)	0.0047 (17)	−0.0039 (17)
C8	0.040 (3)	0.074 (4)	0.027 (3)	0.014 (3)	0.011 (2)	−0.012 (3)
C9	0.0116 (17)	0.0211 (19)	0.025 (2)	−0.0022 (15)	0.0035 (16)	0.0006 (18)
C10	0.018 (2)	0.026 (2)	0.054 (4)	−0.0027 (18)	0.014 (2)	−0.006 (2)
C11	0.022 (2)	0.026 (2)	0.020 (2)	0.0057 (17)	0.0064 (17)	0.0015 (18)
C12	0.062 (4)	0.032 (3)	0.024 (3)	0.012 (2)	0.015 (3)	0.003 (2)
C13	0.024 (2)	0.019 (2)	0.032 (3)	0.0039 (17)	0.012 (2)	0.0024 (19)
C14	0.064 (4)	0.033 (3)	0.052 (4)	0.019 (3)	0.043 (3)	0.019 (3)
C15	0.023 (2)	0.040 (3)	0.021 (2)	0.004 (2)	0.0001 (19)	−0.001 (2)
C16	0.0183 (19)	0.0192 (19)	0.026 (2)	−0.0015 (16)	0.0029 (17)	−0.0016 (18)
O1	0.0160 (13)	0.0196 (14)	0.0253 (17)	−0.0002 (11)	0.0047 (12)	−0.0080 (13)
O2	0.0157 (13)	0.0217 (14)	0.0226 (16)	0.0022 (11)	0.0056 (12)	−0.0054 (13)
O3	0.0208 (14)	0.0266 (16)	0.0227 (17)	−0.0095 (12)	0.0052 (13)	−0.0004 (13)
O4	0.0148 (13)	0.0237 (15)	0.0290 (18)	−0.0010 (12)	0.0041 (13)	0.0070 (14)
O5	0.0144 (13)	0.0203 (14)	0.0305 (18)	0.0012 (11)	0.0126 (13)	0.0046 (13)
O6	0.0194 (14)	0.0187 (14)	0.051 (2)	0.0008 (12)	0.0205 (16)	0.0046 (15)
O7	0.0226 (14)	0.0257 (15)	0.0216 (17)	0.0050 (13)	0.0061 (13)	−0.0046 (13)
O8	0.0152 (13)	0.0310 (16)	0.0188 (16)	0.0012 (12)	0.0033 (12)	−0.0094 (13)
O9	0.0152 (13)	0.0181 (13)	0.0349 (19)	−0.0014 (11)	0.0125 (13)	0.0001 (13)
O10	0.0124 (13)	0.0229 (15)	0.056 (2)	−0.0036 (12)	0.0110 (15)	−0.0136 (16)
O11	0.0317 (16)	0.0180 (14)	0.0242 (17)	0.0077 (13)	0.0102 (14)	0.0021 (13)
O12	0.0335 (17)	0.0223 (15)	0.0192 (17)	0.0019 (13)	0.0007 (14)	0.0001 (13)
O13	0.0225 (14)	0.0140 (13)	0.0364 (19)	0.0029 (11)	0.0152 (14)	0.0046 (13)
O14	0.0321 (17)	0.0192 (14)	0.048 (2)	0.0060 (13)	0.0250 (17)	0.0068 (15)
O15	0.0106 (12)	0.0292 (15)	0.0166 (16)	0.0000 (11)	0.0023 (12)	−0.0009 (13)
O16	0.0158 (15)	0.062 (2)	0.027 (2)	0.0049 (16)	−0.0002 (14)	0.0018 (18)
O17	0.0169 (14)	0.0134 (13)	0.0293 (19)	−0.0001 (12)	−0.0026 (13)	0.0030 (13)
Fe1	0.0098 (2)	0.0123 (2)	0.0186 (3)	0.0006 (2)	0.0016 (2)	−0.0005 (2)
Fe2	0.0119 (2)	0.0124 (2)	0.0203 (3)	0.0004 (2)	0.0067 (2)	0.0002 (2)
Fe3	0.0091 (2)	0.0161 (2)	0.0151 (3)	0.0001 (2)	0.0032 (2)	−0.0013 (2)
N1	0.035 (2)	0.034 (2)	0.039 (3)	−0.0018 (18)	0.016 (2)	0.003 (2)
C17	0.037 (3)	0.060 (4)	0.058 (5)	−0.001 (3)	0.018 (3)	−0.013 (3)
C18	0.047 (3)	0.049 (3)	0.047 (4)	0.001 (3)	0.030 (3)	0.001 (3)
C19	0.054 (3)	0.036 (3)	0.037 (3)	0.013 (2)	0.024 (3)	0.005 (2)
C20	0.073 (5)	0.049 (3)	0.036 (4)	0.014 (3)	0.007 (3)	−0.001 (3)
C21	0.026 (2)	0.055 (3)	0.041 (3)	0.000 (2)	0.016 (2)	0.011 (3)
C22	0.033 (3)	0.043 (3)	0.048 (4)	0.000 (2)	0.017 (3)	0.005 (3)
C23A	0.023 (4)	0.051 (4)	0.054 (7)	−0.018 (4)	0.018 (4)	−0.028 (5)
C24A	0.035 (4)	0.055 (5)	0.040 (6)	−0.011 (4)	0.011 (4)	−0.013 (4)
C25A	0.036 (5)	0.043 (5)	0.062 (8)	−0.001 (4)	0.011 (4)	−0.014 (5)
C26A	0.032 (4)	0.039 (4)	0.060 (7)	−0.007 (4)	0.012 (4)	−0.011 (5)
C27A	0.019 (3)	0.038 (4)	0.058 (6)	−0.010 (3)	0.007 (4)	−0.009 (4)
C28A	0.029 (5)	0.047 (4)	0.055 (6)	−0.014 (3)	0.017 (4)	−0.018 (5)
C29A	0.049 (6)	0.133 (13)	0.094 (12)	0.013 (6)	−0.016 (6)	−0.032 (9)
C23B	0.050 (7)	0.038 (6)	0.060 (9)	−0.012 (5)	0.024 (6)	−0.018 (6)
C24B	0.039 (6)	0.042 (6)	0.071 (11)	0.002 (5)	0.028 (7)	−0.012 (7)
C25B	0.049 (8)	0.032 (7)	0.082 (13)	−0.006 (5)	0.021 (9)	−0.010 (8)
C26B	0.042 (7)	0.042 (6)	0.063 (11)	−0.011 (5)	0.023 (7)	−0.007 (7)
C27B	0.051 (7)	0.035 (6)	0.045 (8)	−0.004 (5)	0.026 (6)	−0.011 (6)
C28B	0.060 (8)	0.035 (7)	0.058 (11)	−0.012 (5)	0.012 (9)	0.001 (7)
C29B	0.062 (8)	0.066 (10)	0.038 (9)	−0.006 (7)	0.004 (7)	0.010 (8)
O18	0.0109 (12)	0.0149 (12)	0.0165 (15)	0.0006 (10)	0.0037 (11)	−0.0011 (11)

### Geometric parameters (Å, °)

**Table:**

C1—O1	1.256 (5)	Fe2—O13	2.027 (3)
C1—O2	1.256 (5)	Fe3—O15	1.995 (3)
C1—C2	1.498 (6)	Fe1—O17	2.045 (3)
C2—H2A	0.9800	O17—H10	0.825 (16)
C2—H2B	0.9800	O17—H11	0.833 (16)
C2—H2C	0.9800	Fe1—O18	1.906 (3)
C3—O4	1.253 (5)	Fe2—O18	1.924 (3)
C3—O3	1.257 (6)	Fe3—O18	1.945 (3)
C3—C4	1.503 (7)	N1—C21	1.487 (7)
C4—H4A	0.9800	N1—C19	1.519 (7)
C4—H4B	0.9800	N1—C18	1.524 (8)
C4—H4C	0.9800	N1—H1	0.9300
C5—O6	1.252 (5)	C17—C18	1.488 (9)
C5—O5	1.259 (5)	C17—H17A	0.9800
C5—C6	1.513 (6)	C17—H17B	0.9800
C6—H6A	0.9800	C17—H17C	0.9800
C6—H6B	0.9800	C18—H18A	0.9900
C6—H6C	0.9800	C18—H18B	0.9900
C7—O8	1.256 (5)	C19—C20	1.461 (9)
C7—O7	1.260 (5)	C19—H19A	0.9900
C7—C8	1.508 (7)	C19—H19B	0.9900
C8—H8A	0.9800	C20—H20A	0.9800
C8—H8B	0.9800	C20—H20B	0.9800
C8—H8C	0.9800	C20—H20C	0.9800
C9—O10	1.248 (5)	C21—C22	1.524 (8)
C9—O9	1.257 (5)	C21—H21A	0.9900
C9—C10	1.515 (5)	C21—H21B	0.9900
C10—H10A	0.9800	C22—H22A	0.9800
C10—H10B	0.9800	C22—H22B	0.9800
C10—H10C	0.9800	C22—H22C	0.9800
C11—O11	1.249 (5)	C23A—C24A	1.361 (13)
C11—O12	1.261 (5)	C23A—C28A	1.374 (16)
C11—C12	1.504 (7)	C23A—H23A	0.9500
C12—H12A	0.9800	C24A—C25A	1.390 (12)
C12—H12B	0.9800	C24A—C29A	1.483 (17)
C12—H12C	0.9800	C25A—C26A	1.376 (13)
C13—O13	1.255 (5)	C25A—H25A	0.9500
C13—O14	1.270 (5)	C26A—C27A	1.375 (12)
C13—C14	1.498 (7)	C26A—H26A	0.9500
C14—H14A	0.9800	C27A—C28A	1.392 (11)
C14—H14B	0.9800	C27A—H27A	0.9500
C14—H14C	0.9800	C28A—H28A	0.9500
C15—C16	1.491 (7)	C29A—H29A	0.9800
C15—H15A	0.9800	C29A—H29B	0.9800
C15—H15B	0.9800	C29A—H29C	0.9800
C15—H15C	0.9800	C23B—C24B	1.362 (15)
C16—O16	1.239 (5)	C23B—C28B	1.38 (2)
C16—O15	1.280 (6)	C23B—H23B	0.9500
Fe1—O1	2.026 (3)	C24B—C25B	1.393 (14)
Fe3—O2	2.031 (3)	C24B—H24B	0.9500
Fe1—O3	2.030 (3)	C25B—C26B	1.374 (17)
Fe3—O4	2.012 (3)	C25B—H25B	0.9500
Fe3—O5	2.017 (3)	C26B—C27B	1.391 (14)
Fe2—O6	2.021 (3)	C26B—H26B	0.9500
Fe2—O7	2.032 (3)	C27B—C28B	1.372 (16)
Fe3—O8	2.017 (3)	C27B—C29B	1.44 (3)
Fe2—O9	2.028 (3)	C28B—H28B	0.9500
Fe1—O10	2.026 (3)	C29B—H29D	0.9800
Fe2—O11	2.018 (4)	C29B—H29E	0.9800
Fe1—O12	2.029 (3)	C29B—H29F	0.9800
			
O1—C1—O2	125.4 (4)	O11—Fe2—O9	90.93 (14)
O1—C1—C2	117.7 (4)	O6—Fe2—O9	167.59 (12)
O2—C1—C2	116.9 (3)	O13—Fe2—O9	81.36 (11)
C1—C2—H2A	109.5	O18—Fe2—O7	92.95 (12)
C1—C2—H2B	109.5	O11—Fe2—O7	174.95 (12)
H2A—C2—H2B	109.5	O6—Fe2—O7	92.87 (15)
C1—C2—H2C	109.5	O13—Fe2—O7	85.96 (13)
H2A—C2—H2C	109.5	O9—Fe2—O7	85.65 (13)
H2B—C2—H2C	109.5	O18—Fe3—O15	178.60 (13)
O4—C3—O3	125.7 (4)	O18—Fe3—O4	90.23 (12)
O4—C3—C4	116.7 (4)	O15—Fe3—O4	88.43 (12)
O3—C3—C4	117.6 (4)	O18—Fe3—O5	95.17 (12)
C3—C4—H4A	109.5	O15—Fe3—O5	86.18 (12)
C3—C4—H4B	109.5	O4—Fe3—O5	174.50 (12)
H4A—C4—H4B	109.5	O18—Fe3—O8	92.16 (11)
C3—C4—H4C	109.5	O15—Fe3—O8	87.44 (12)
H4A—C4—H4C	109.5	O4—Fe3—O8	88.47 (14)
H4B—C4—H4C	109.5	O5—Fe3—O8	92.36 (13)
O6—C5—O5	125.3 (4)	O18—Fe3—O2	96.56 (12)
O6—C5—C6	117.6 (4)	O15—Fe3—O2	83.84 (12)
O5—C5—C6	117.0 (4)	O4—Fe3—O2	91.27 (13)
C5—C6—H6A	109.5	O5—Fe3—O2	87.10 (12)
C5—C6—H6B	109.5	O8—Fe3—O2	171.28 (12)
H6A—C6—H6B	109.5	C21—N1—C19	113.2 (4)
C5—C6—H6C	109.5	C21—N1—C18	113.1 (4)
H6A—C6—H6C	109.5	C19—N1—C18	109.1 (4)
H6B—C6—H6C	109.5	C21—N1—H1	107.0
O8—C7—O7	125.1 (4)	C19—N1—H1	107.0
O8—C7—C8	116.3 (4)	C18—N1—H1	107.0
O7—C7—C8	118.6 (4)	C18—C17—H17A	109.5
C7—C8—H8A	109.5	C18—C17—H17B	109.5
C7—C8—H8B	109.5	H17A—C17—H17B	109.5
H8A—C8—H8B	109.5	C18—C17—H17C	109.5
C7—C8—H8C	109.5	H17A—C17—H17C	109.5
H8A—C8—H8C	109.5	H17B—C17—H17C	109.5
H8B—C8—H8C	109.5	C17—C18—N1	109.7 (5)
O10—C9—O9	124.7 (4)	C17—C18—H18A	109.7
O10—C9—C10	117.4 (4)	N1—C18—H18A	109.7
O9—C9—C10	117.9 (4)	C17—C18—H18B	109.7
C9—C10—H10A	109.5	N1—C18—H18B	109.7
C9—C10—H10B	109.5	H18A—C18—H18B	108.2
H10A—C10—H10B	109.5	C20—C19—N1	112.1 (5)
C9—C10—H10C	109.5	C20—C19—H19A	109.2
H10A—C10—H10C	109.5	N1—C19—H19A	109.2
H10B—C10—H10C	109.5	C20—C19—H19B	109.2
O11—C11—O12	125.5 (4)	N1—C19—H19B	109.2
O11—C11—C12	117.5 (4)	H19A—C19—H19B	107.9
O12—C11—C12	117.0 (4)	C19—C20—H20A	109.5
C11—C12—H12A	109.5	C19—C20—H20B	109.5
C11—C12—H12B	109.5	H20A—C20—H20B	109.5
H12A—C12—H12B	109.5	C19—C20—H20C	109.5
C11—C12—H12C	109.5	H20A—C20—H20C	109.5
H12A—C12—H12C	109.5	H20B—C20—H20C	109.5
H12B—C12—H12C	109.5	N1—C21—C22	113.4 (4)
O13—C13—O14	121.3 (4)	N1—C21—H21A	108.9
O13—C13—C14	120.1 (4)	C22—C21—H21A	108.9
O14—C13—C14	118.6 (4)	N1—C21—H21B	108.9
C13—C14—H14A	109.5	C22—C21—H21B	108.9
C13—C14—H14B	109.5	H21A—C21—H21B	107.7
H14A—C14—H14B	109.5	C21—C22—H22A	109.5
C13—C14—H14C	109.5	C21—C22—H22B	109.5
H14A—C14—H14C	109.5	H22A—C22—H22B	109.5
H14B—C14—H14C	109.5	C21—C22—H22C	109.5
C16—C15—H15A	109.5	H22A—C22—H22C	109.5
C16—C15—H15B	109.5	H22B—C22—H22C	109.5
H15A—C15—H15B	109.5	C24A—C23A—C28A	120.5 (9)
C16—C15—H15C	109.5	C24A—C23A—H23A	119.7
H15A—C15—H15C	109.5	C28A—C23A—H23A	119.7
H15B—C15—H15C	109.5	C23A—C24A—C25A	120.4 (10)
O16—C16—O15	121.0 (4)	C23A—C24A—C29A	116.5 (11)
O16—C16—C15	119.3 (4)	C25A—C24A—C29A	123.1 (13)
O15—C16—C15	119.7 (4)	C26A—C25A—C24A	119.5 (10)
C1—O1—Fe1	134.5 (3)	C26A—C25A—H25A	120.3
C1—O2—Fe3	128.5 (3)	C24A—C25A—H25A	120.3
C3—O3—Fe1	126.8 (3)	C27A—C26A—C25A	119.9 (9)
C3—O4—Fe3	133.2 (3)	C27A—C26A—H26A	120.0
C5—O5—Fe3	129.4 (2)	C25A—C26A—H26A	120.0
C5—O6—Fe2	136.5 (3)	C26A—C27A—C28A	120.3 (9)
C7—O7—Fe2	129.3 (3)	C26A—C27A—H27A	119.9
C7—O8—Fe3	135.0 (3)	C28A—C27A—H27A	119.9
C9—O9—Fe2	131.1 (3)	C23A—C28A—C27A	119.2 (9)
C9—O10—Fe1	134.4 (3)	C23A—C28A—H28A	120.4
C11—O11—Fe2	135.1 (3)	C27A—C28A—H28A	120.4
C11—O12—Fe1	129.5 (3)	C24B—C23B—C28B	119.9 (13)
C13—O13—Fe2	143.2 (3)	C24B—C23B—H23B	120.1
C16—O15—Fe3	143.4 (3)	C28B—C23B—H23B	120.1
Fe1—O17—H10	123 (4)	C23B—C24B—C25B	120.0 (15)
Fe1—O17—H11	122 (4)	C23B—C24B—H24B	120.0
H10—O17—H11	112 (5)	C25B—C24B—H24B	120.0
O18—Fe1—O1	98.04 (11)	C26B—C25B—C24B	118.2 (14)
O18—Fe1—O10	98.97 (12)	C26B—C25B—H25B	120.9
O1—Fe1—O10	162.95 (12)	C24B—C25B—H25B	120.9
O18—Fe1—O12	92.25 (13)	C25B—C26B—C27B	123.2 (14)
O1—Fe1—O12	91.26 (13)	C25B—C26B—H26B	118.4
O10—Fe1—O12	89.39 (15)	C27B—C26B—H26B	118.4
O18—Fe1—O3	91.25 (12)	C28B—C27B—C26B	116.0 (14)
O1—Fe1—O3	91.14 (13)	C28B—C27B—C29B	120.1 (15)
O10—Fe1—O3	87.19 (14)	C26B—C27B—C29B	123.8 (16)
O12—Fe1—O3	175.45 (13)	C27B—C28B—C23B	122.2 (14)
O18—Fe1—O17	179.11 (13)	C27B—C28B—H28B	118.9
O1—Fe1—O17	81.14 (12)	C23B—C28B—H28B	118.9
O10—Fe1—O17	81.87 (12)	C27B—C29B—H29D	109.5
O12—Fe1—O17	87.44 (14)	C27B—C29B—H29E	109.5
O3—Fe1—O17	89.10 (13)	H29D—C29B—H29E	109.5
O18—Fe2—O11	91.22 (12)	C27B—C29B—H29F	109.5
O18—Fe2—O6	93.91 (11)	H29D—C29B—H29F	109.5
O11—Fe2—O6	89.68 (15)	H29E—C29B—H29F	109.5
O18—Fe2—O13	178.91 (14)	Fe1—O18—Fe2	119.34 (14)
O11—Fe2—O13	89.86 (13)	Fe1—O18—Fe3	119.94 (13)
O6—Fe2—O13	86.25 (11)	Fe2—O18—Fe3	120.71 (14)
O18—Fe2—O9	98.48 (11)		
			
O2—C1—O1—Fe1	4.9 (7)	C16—O15—Fe3—O5	137.6 (5)
C2—C1—O1—Fe1	−175.3 (3)	C16—O15—Fe3—O8	45.1 (5)
O1—C1—O2—Fe3	19.3 (6)	C16—O15—Fe3—O2	−134.9 (5)
C2—C1—O2—Fe3	−160.5 (3)	C3—O4—Fe3—O18	−5.0 (4)
O4—C3—O3—Fe1	−12.3 (7)	C3—O4—Fe3—O15	174.6 (4)
C4—C3—O3—Fe1	166.7 (4)	C3—O4—Fe3—O5	−174.1 (13)
O3—C3—O4—Fe3	36.5 (7)	C3—O4—Fe3—O8	87.1 (4)
C4—C3—O4—Fe3	−142.5 (4)	C3—O4—Fe3—O2	−101.6 (4)
O6—C5—O5—Fe3	−6.5 (7)	C5—O5—Fe3—O18	33.2 (4)
C6—C5—O5—Fe3	171.2 (3)	C5—O5—Fe3—O15	−146.5 (4)
O5—C5—O6—Fe2	−11.0 (8)	C5—O5—Fe3—O4	−157.8 (13)
C6—C5—O6—Fe2	171.2 (4)	C5—O5—Fe3—O8	−59.2 (4)
O8—C7—O7—Fe2	5.5 (6)	C5—O5—Fe3—O2	129.5 (4)
C8—C7—O7—Fe2	−173.8 (4)	C7—O8—Fe3—O18	−0.2 (4)
O7—C7—O8—Fe3	−25.2 (7)	C7—O8—Fe3—O15	−178.9 (4)
C8—C7—O8—Fe3	154.1 (4)	C7—O8—Fe3—O4	−90.4 (4)
O10—C9—O9—Fe2	6.5 (7)	C7—O8—Fe3—O5	95.1 (4)
C10—C9—O9—Fe2	−171.1 (3)	C7—O8—Fe3—O2	−178.7 (7)
O9—C9—O10—Fe1	8.4 (8)	C1—O2—Fe3—O18	−41.0 (4)
C10—C9—O10—Fe1	−174.1 (4)	C1—O2—Fe3—O15	137.7 (4)
O12—C11—O11—Fe2	11.4 (7)	C1—O2—Fe3—O4	49.4 (4)
C12—C11—O11—Fe2	−169.5 (3)	C1—O2—Fe3—O5	−135.9 (4)
O11—C11—O12—Fe1	−0.4 (7)	C1—O2—Fe3—O8	137.5 (8)
C12—C11—O12—Fe1	−179.5 (3)	C21—N1—C18—C17	59.5 (6)
O14—C13—O13—Fe2	179.2 (3)	C19—N1—C18—C17	−173.6 (4)
C14—C13—O13—Fe2	0.7 (8)	C21—N1—C19—C20	−63.6 (6)
O16—C16—O15—Fe3	175.1 (4)	C18—N1—C19—C20	169.5 (5)
C15—C16—O15—Fe3	−1.8 (7)	C19—N1—C21—C22	125.8 (5)
C1—O1—Fe1—O18	1.8 (4)	C18—N1—C21—C22	−109.5 (5)
C1—O1—Fe1—O10	−173.7 (5)	C28A—C23A—C24A—C25A	−4.0 (14)
C1—O1—Fe1—O12	94.3 (4)	C28A—C23A—C24A—C29A	174.6 (13)
C1—O1—Fe1—O3	−89.6 (4)	C23A—C24A—C25A—C26A	0.2 (14)
C1—O1—Fe1—O17	−178.5 (4)	C29A—C24A—C25A—C26A	−178.3 (15)
C9—O10—Fe1—O18	5.4 (5)	C24A—C25A—C26A—C27A	2.5 (19)
C9—O10—Fe1—O1	−179.1 (4)	C25A—C26A—C27A—C28A	−1.4 (18)
C9—O10—Fe1—O12	−86.8 (5)	C24A—C23A—C28A—C27A	5.1 (18)
C9—O10—Fe1—O3	96.2 (5)	C26A—C27A—C28A—C23A	−2.4 (17)
C9—O10—Fe1—O17	−174.3 (5)	C28B—C23B—C24B—C25B	−4.2 (18)
C11—O12—Fe1—O18	−30.8 (4)	C23B—C24B—C25B—C26B	−1.7 (16)
C11—O12—Fe1—O1	−128.9 (4)	C24B—C25B—C26B—C27B	3(3)
C11—O12—Fe1—O10	68.2 (4)	C25B—C26B—C27B—C28B	2(3)
C11—O12—Fe1—O3	109.4 (16)	C25B—C26B—C27B—C29B	178 (2)
C11—O12—Fe1—O17	150.1 (4)	C26B—C27B—C28B—C23B	−8(4)
C3—O3—Fe1—O18	−31.3 (4)	C29B—C27B—C28B—C23B	176 (2)
C3—O3—Fe1—O1	66.8 (4)	C24B—C23B—C28B—C27B	9(3)
C3—O3—Fe1—O10	−130.2 (4)	O1—Fe1—O18—Fe2	149.07 (16)
C3—O3—Fe1—O12	−171.4 (15)	O10—Fe1—O18—Fe2	−32.25 (19)
C3—O3—Fe1—O17	147.9 (4)	O12—Fe1—O18—Fe2	57.48 (17)
C11—O11—Fe2—O18	13.6 (4)	O3—Fe1—O18—Fe2	−119.60 (17)
C11—O11—Fe2—O6	107.5 (4)	O17—Fe1—O18—Fe2	127 (9)
C11—O11—Fe2—O13	−166.2 (4)	O1—Fe1—O18—Fe3	−31.10 (18)
C11—O11—Fe2—O9	−84.9 (4)	O10—Fe1—O18—Fe3	147.57 (18)
C11—O11—Fe2—O7	−132.1 (14)	O12—Fe1—O18—Fe3	−122.69 (17)
C5—O6—Fe2—O18	−5.9 (5)	O3—Fe1—O18—Fe3	60.22 (17)
C5—O6—Fe2—O11	−97.1 (5)	O17—Fe1—O18—Fe3	−53 (9)
C5—O6—Fe2—O13	173.0 (5)	O11—Fe2—O18—Fe1	−51.24 (17)
C5—O6—Fe2—O9	170.0 (6)	O6—Fe2—O18—Fe1	−141.00 (18)
C5—O6—Fe2—O7	87.3 (5)	O13—Fe2—O18—Fe1	121 (6)
C13—O13—Fe2—O18	142 (6)	O9—Fe2—O18—Fe1	39.88 (19)
C13—O13—Fe2—O11	−45.9 (5)	O7—Fe2—O18—Fe1	125.92 (17)
C13—O13—Fe2—O6	43.8 (5)	O11—Fe2—O18—Fe3	128.94 (17)
C13—O13—Fe2—O9	−136.9 (5)	O6—Fe2—O18—Fe3	39.17 (19)
C13—O13—Fe2—O7	136.9 (5)	O13—Fe2—O18—Fe3	−59 (6)
C9—O9—Fe2—O18	−29.3 (4)	O9—Fe2—O18—Fe3	−139.94 (17)
C9—O9—Fe2—O11	62.0 (4)	O7—Fe2—O18—Fe3	−53.91 (17)
C9—O9—Fe2—O6	154.8 (6)	O15—Fe3—O18—Fe1	−63 (6)
C9—O9—Fe2—O13	151.8 (4)	O4—Fe3—O18—Fe1	−47.54 (18)
C9—O9—Fe2—O7	−121.7 (4)	O5—Fe3—O18—Fe1	131.42 (17)
C7—O7—Fe2—O18	30.2 (4)	O8—Fe3—O18—Fe1	−136.02 (17)
C7—O7—Fe2—O11	175.9 (14)	O2—Fe3—O18—Fe1	43.76 (18)
C7—O7—Fe2—O6	−63.9 (4)	O15—Fe3—O18—Fe2	117 (5)
C7—O7—Fe2—O13	−149.9 (4)	O4—Fe3—O18—Fe2	132.28 (17)
C7—O7—Fe2—O9	128.5 (4)	O5—Fe3—O18—Fe2	−48.76 (18)
C16—O15—Fe3—O18	−28 (6)	O8—Fe3—O18—Fe2	43.81 (18)
C16—O15—Fe3—O4	−43.5 (5)	O2—Fe3—O18—Fe2	−136.42 (16)

### Hydrogen-bond geometry (Å, °)

**Table:**

*D*—H···*A*	*D*—H	H···*A*	*D*···*A*	*D*—H···*A*
N1—H1···O14^i^	0.93	1.87	2.789 (6)	168.
O17—H10···O16^ii^	0.83 (2)	1.81 (2)	2.635 (5)	176 (5)
O17—H11···O14^iii^	0.83 (2)	1.83 (2)	2.665 (4)	178 (5)
C12—H12B···O7^iv^	0.98	2.55	3.526 (6)	174
C14—H14C···O6	0.98	2.54	3.166 (7)	122
C15—H15C···O8	0.98	2.53	3.188 (6)	125
C19—H19A···O16^v^	0.99	2.37	3.221 (6)	144
C21—H21B···O15^v^	0.99	2.55	3.418 (5)	146
C22—H22A···O3^vi^	0.98	2.55	3.476 (6)	157

Symmetry codes: (i) *x*−1/2, *y*−1/2, *z*; (ii) *x*−1/2, −*y*+3/2, *z*−1/2; (iii) *x*, *y*−1, *z*; (iv) *x*, −*y*+2, *z*−1/2; (v) *x*−1, *y*, *z*; (vi) *x*−1/2, *y*+1/2, *z*.
